# The role of car tyres in the ecology of *Aedes aegypti* mosquitoes in Ghana

**DOI:** 10.1016/j.crpvbd.2024.100176

**Published:** 2024-04-30

**Authors:** Anisa Abdulai, Christopher Mfum Owusu-Asenso, Christodea Haizel, Sebastian Kow Egyin Mensah, Isaac Kwame Sraku, Daniel Halou, Richard Tettey Doe, Abdul Rahim Mohammed, Yaw Akuamoah-Boateng, Akua Obeng Forson, Yaw Asare Afrane

**Affiliations:** a Centre for Vector-Borne Disease Research, Department of Medical Microbiology, University of Ghana Medical School, University of Ghana, Legon, Ghana; b Department of Medical Laboratory Sciences, School of Biomedical and Allied Health Sciences, University of Ghana, Accra, Ghana

**Keywords:** *Aedes aegypti*, Life tables, Oviposition, Ecology, Larval survivorship

## Abstract

*Aedes aegypti* is an important vector of arboviral diseases including dengue and yellow fever. Despite the wide distribution of this mosquito species, there are limited data on the ecology of *Ae*. *aegypti* in Ghana. In this study, we report on the oviposition preference and the larval life tables of *Ae*. *aegypti* mosquitoes in Accra, Ghana. The oviposition preference of the mosquitoes to three habitat types (car tyres, drums and bowls) was measured by setting up ovitraps. We recorded the presence and abundance of larvae every 3 days. Two-hour-old *Ae. aegypti* larvae were introduced and raised in three habitat types to undertake larval life tables. The number of surviving larvae at each developmental stage was recorded daily until they emerged as adults. Car tyres showed a higher abundance of *Ae. aegypti* larvae (52.3%) than drums (32.5%) and bowls (15.1%) (ANOVA, *F*_(2,__159)_ = 18.79, *P* < 0.001). The mean development time of *Ae. aegypti* larvae was significantly lower in car tyres (7 ± 1 days) compared to that of bowls (9 ± 0.0 days) and drums (12.6 ± 1.5 days) (*P* = 0.024). The differences in pupation rates and emergence rates were not significant across the habitat types; however, the highest pupation rate was observed in bowls (0.92 ± 0.17) and the emergence rate was highest in tyres (0.84 ± 0.10). The proportion of first-instar larvae that survived to emergence was significantly higher in car tyres (0.84 ± 0.10) compared to that of bowls (0.72 ± 0.20) and drums (0.62 ± 0.20) (*P* = 0.009). No mortalities were observed after 9 days in car tyres, 10 days in bowls and 15 days in drums. The results confirm that discarded car tyres were the preferred habitat choice for the oviposition of gravid female *Ae*. *aegypti* mosquitoes and provide the best habitat conditions for larval development and survival. These findings are necessary for understanding the ecology of *Ae. aegypti* to develop appropriate strategies for their control in Ghana.

## Highlights

• Higher *Aedes aegypti* larval abundance was observed in car tyres compared to bowls and drums.
• This indicates that car tyres are the preferred choice for oviposition for gravid female *Ae. aegypti* mosquitoes.
• Shorter larval development time and higher survival were observed in car tyres than in other habitat types (bowls and drums).
• There was no significant difference in pupation and emergence rates of *Ae. aegypti* larvae in car tyres, bowls and drums.

## Graphical abstract

**Figure:**
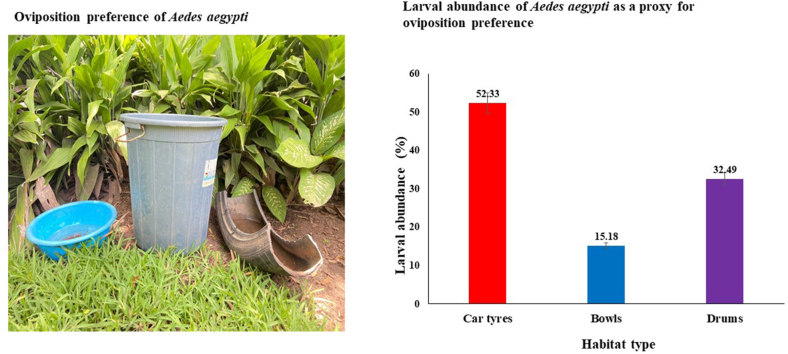

## 1 Introduction

Yellow fever and dengue fever outbreaks have become more frequent in West Africa in the last five years (Suzuki et al., 2017; Amoako et al., 2018; Tarnagda et al., 2018; Weetman et al., 2018; Adogo and Ogoh, 2020; WHO, 2021). *Aedes aegypti* mosquitoes transmit arboviruses that cause diseases such as yellow fever, dengue fever, Zika, chikungunya and Rift Valley fever. *Aedes aegypti* is highly anthropophilic and mainly adapted to urban settings (Powell and Tabachnick, 2013). Domestic forms of African *Ae. aegypti* are more adapted to breeding in artificial containers close to human settlements (Brown et al., 2011; Crawford et al., 2017). However, the sylvatic forms of *Ae. aegypti* breed in natural breeding habitats such as rock pools, tree holes and fruit husks in forested areas (Powell and Tabachnick, 2013). The main vector control strategies for *Aedes* spp. are chemical interventions using insecticides and larval source management (Weeratunga et al., 2017).

The World Health Organization (WHO) recommends using larval source management (LSM) to control the immature stages of *Aedes* spp. mosquitoes (WHO, 2012). This will help in reducing the densities of these vectors within communities. Currently, evidence of insecticide resistance to the four main insecticides in adult *Ae. aegypti* has been detected in Ghana and across West Africa (Badolo et al., 2019; Konan et al., 2021; Kwame Amlalo et al., 2022; Owusu-Asenso et al., 2022; Toé et al., 2022). Controlling the immature stages of *Aedes* spp. using LSM helps reduce the dependence on the main insecticides by combining larviciding and habitat modifications and manipulation (WHO, 2013; Adetoro et al., 2022).

To control the immature stages of *Aedes* spp., it is critical to understand the behaviour of these vectors such as their preferred habitat for oviposition and their life history traits. Female *Ae*. *aegypti* in urban and suburban areas prefer to breed in artificial, man-made containers such as tyres, discarded containers, flower pots or drums (Maciel-de-Freitas et al., 2007). *Aedes aegypti* from different areas have been found to exhibit different oviposition preferences (Simard et al., 2005; Zahouli et al., 2016; Zuharah et al., 2016; Egid et al., 2022). Several studies in West Africa including Ghana have found *Ae*. *aegypti* breeding predominately in car tyres (Kamgang et al., 2013; Kudom, 2020; Tedjou et al., 2020; Wat'senga Tezzo et al., 2021; Owusu-Asenso et al., 2022). Tyres are especially useful for mosquito reproduction because they are mostly stored outdoors and can collect and maintain rainwater for a long period. Moreover, car tyres serve as an excellent breeding habitat for *Aedes* spp. mosquitoes because decaying leaves from neighbouring trees provide chemical conditions similar to tree holes (Kweka et al., 2018). However, other studies have also found *Ae. aegypti* larvae to breed predominately in other habitat types such as large water barrels, animal troughs, plastic containers and septic tanks (Kweka et al., 2018; Kampango et al., 2021; Badolo et al., 2022).

A female mosquito’s choice of habitat type and oviposition may be influenced by several factors such as the season, habitat size and nutritional availability (Spencer et al., 2005; Maciel-de-Freitas et al., 2007). Female mosquitoes increase the survival and development of their offspring by selecting breeding habitats that reduce the risks of predators and competition (Blaustein et al., 2004; Wong et al., 2011). For instance, *Ae. aegypti* mostly rest and feed outdoors, so they can explore a wider range of habitat types that are far from human settlements (Wong et al., 2011). Several studies in Ghana found *Aedes* spp. abundance to be significantly higher outdoors than indoors (Captain-Esoah et al., 2020; Owusu-Asenso et al., 2022). Understanding the oviposition choices of female *Aedes* spp. mosquitoes will help in designing targeted LSM interventions for these vectors.

Mosquito larval development and survivorship are important determinants of vector densities within an area (Li et al., 2014). Several studies have found that there are variations in life history traits such as larval development and survival in *Ae. aegypti* in different areas (Bong et al., 2021; Cui et al., 2021). This is because different areas may have diverse environmental conditions which may affect larval development, survival and adult emergence (Afrane et al., 2008; Yang et al., 2020). Understanding the life history traits of *Ae*. *aegypti* in Ghana is very important for the development of appropriate vector control measures for the immature stages of these vectors. This study aims to investigate the oviposition preference and the larval life history traits of *Ae. aegypti* in Accra, Ghana. An experimental study was conducted to determine the oviposition preference and life history traits in three different habitat types (car tyres, bowls and drums). This information will greatly improve vector control strategies especially larval source management for arboviral disease vectors in Ghana.

## 2 Materials and methods

### 2.1 Study site

The study was conducted within Korle-bu (5°33′N, 0°12′W), Accra, Ghana, from November 2020 to March 2021. Korle-bu is a suburb located in the coastal savannah zone of the Greater Accra Region. This area has the largest teaching and referral hospital in Ghana and therefore it has an influx of people from different regions across Ghana. This is a populous cosmopolitan area in Accra with numerous vulcanizing shops (car tyre repair shops) within the area. The inhabitants also tend to store water in storage containers due to the irregular flow of pipe-borne water. There is an abundance of *Aedes* spp. breeding habitats ranging mostly from used car tyres, small containers and water storage drums.

### 2.2 *Aedes aegypti* oviposition in three habitat types

An oviposition experiment was set up to investigate the preferred habitat type for *Ae. aegypti* using three habitat types, i.e. car tyres, bowls and drums. Car tyre ovitraps were made by cutting a car tyre into three parts that can hold water. Each car tyre ovitrap was approximately 56 cm in length and 15 cm in height with an aperture of 5 cm. Bowls were plastic, 29 cm in diameter and 8 cm in height, and had a capacity of up to 5 l of water. Drums were plastic, 41 cm in diameter and 42 cm in height, and had a water capacity of 50 l. The bowls and drums were dark-coloured (black, grey and blue).

Each container type was filled with 2–5 l of rainwater. The containers were placed under trees with a canopy cover of about 50% to mimic where *Ae. aegypti* habitats were found in previous surveys (Owusu-Asenso et al., 2022; Abdulai et al., 2023). For the reproducibility of the experiment, 3 replicates of each habitat type were set up in 3 different spots (700–900 m apart). The spots where the replicates were set up were about 15–20 m from human dwellings. At each area, all three different habit types were set up 4–5 m apart to determine which type was preferred for oviposition by gravid female *Ae. aegypti* mosquitoes.

Larval abundance was used as a proxy for oviposition preference because of the difficulty in identifying and counting the eggs that were laid in the different habitats, especially tyres because of their dark surface. The replicates were checked for the presence of larvae every three days for 4 months (from December 2020 to March 2021). The number of larvae was recorded for each habitat type. The larvae were removed, and the water was replaced after every recording.

### 2.3 Larval life table in three habitat types

#### 2.3.1 F1 eggs for *Ae. aegypti* larval life table

*Aedes aegypti* larvae were collected from breeding habitats in Korle-bu and transported to the insectary at the University of Ghana Medical School, Accra. In the insectary, the larvae were raised into adults. Adult female *Ae. aegypti* were blood-fed with a membrane feeder. After 48 h, egg-laying pads for oviposition by gravid females were prepared with damp cotton in a Petri dish lined with filter paper and placed in the cages. First filial generation (F1) eggs obtained were washed into larval bowls containing rainwater.

#### 2.3.2 *Aedes aegypti* larval life-table experiments

Life-table experiments were performed to determine the life history traits of *Ae*. *aegypti* in different habitat types in November 2020. The life-table components investigated were larval development time, pupation rates, emergence rate and sex ratio. Experiments were set up in 3 replicates each, for 3 different habitat types, i.e. car tyres, drums and bowls. For each replicate, 30 2-h-old larvae were introduced into each habitat type, containing 2–5 l of rainwater and covered with a muslin net. Each habitat type was placed under trees in gardens and outdoors of human dwelling houses. The replicates were observed daily, and the volume of water was replenished daily to compensate for loss due to evaporation.

Developmental stages of surviving *Ae. aegypti* larvae were assessed daily; alive and dead larvae were recorded. Pupae collected from the habitat types were held in pre-labelled individual paper cups with water of a depth of 25 ml for adult emergence. The paper cups containing one pupa per cup were covered with muslin netting and observed daily for adult emergence. Pupal mortality, adult mosquitoes that emerged (emergence rate), and the sexes were recorded daily. When pupation started, habitats were visited twice a day, at 8:00 h and 17:00 h, for pupae collections.

### 2.4 Data analysis

Larval abundance was calculated as the total number of larvae obtained per habitat type over the total number of larvae collected. This was used to measure the oviposition preference of *Ae. aegypti* female mosquitoes to the three habitat types. For the larval life-table experiment, the larval development time was recorded in days as the duration from the first-instar larval stage to the pupal stage. Mean larval development time was defined as the average duration, in days, of first-instar larvae to develop into pupae. The pupation rate was calculated as the sum of the total number of pupae per the sum of the total number of first-instar larvae. The emergence rate was calculated as the sum of the number of adults that emerged per the total number of pupae. Survivorship of *Ae. aegypti* larvae was calculated as the proportion of first-instar larvae that survived to adults. The ratio of females to males was determined by counting and recording the number of males and females that emerged per day over the total number of adults that emerged. Histograms with normal distribution curves and boxplots were used to assess the normality of the datasets. ANOVA and Kruskal-Wallis ranked sum test were used to test the statistical significance of the different habitat types on larval survivorship and larval development time wherever appropriate for normally distributed and skewed data respectively. Dunn’s test was used to compare the significance of the means. Pearson Chi-square test was used to test for significance in survivorship. All data analyses were performed using R version 3.6.3.

Air temperature and relative humidity data during the period of the experiments were obtained from the ECMWF Reanalysis v5. (ERA5 climate data) (ECMWF Reanalysis v5, 2018). ERA5 provides hourly estimates of a large number of atmospheric, land and oceanic climate variables. Generalized linear mixed model (GLMM) was used to determine the relationships and interactions between the environmental climatic conditions (air temperature and humidity), replication and type of breeding containers on larval abundance and larval life-table parameters of *Ae*. *aegypti*.

## 3 Results

### 3.1 Variations in the experimental conditions

Air temperature during the oviposition experiments (December to March) and the larval life-table experiments (November) is shown in Table 1. The mean temperatures during the oviposition experiments ranged between 27.7 °C and 28.7 °C, and the mean relative humidity ranged from 73.3% to 82.4%. For the larval life-table experiments, the mean temperature and relative humidity were 27.7 °C and 80%, respectively.

**Table 1:** Air temperature and relative humidity during the oviposition and larval life-table experiments.

Month	Temperature (°C) (Mean ± SD)	Relative humidity (%) (Mean ± SD)
November	27.72 ± 0.35	80.0 ± 0.10
December	27.74 ± 0.32	73.3 ± 0.05
January	27.71 ± 0.42	78.6 ± 0.20
February	28.42 ± 0.33	81.2 ± 0.15
March	28.70 ± 0.88	82.4 ± 0.31

*Abbreviation*: SD, standard deviation.

### 3.2 Abundance of immature *Ae*. *aegypti* in three different habitat types

**Fig. 1:**
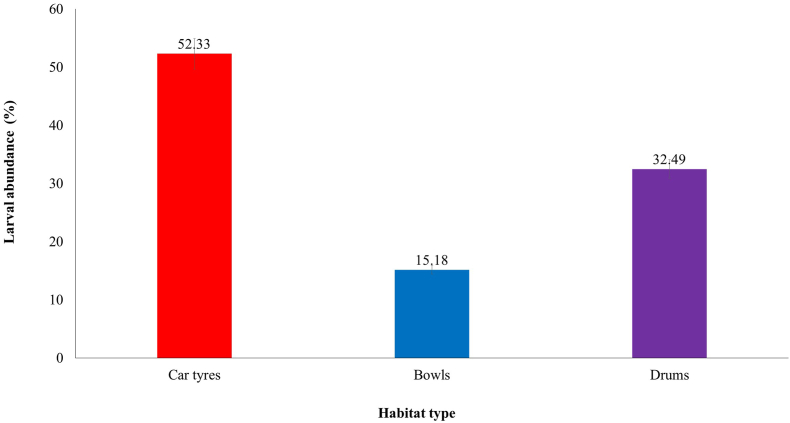
Abundance of immature *Aedes aegypti* collected from different habitat types (tyres, bowls and drums). Error bars represent the 95% confidence intervals.

A total of 4059 immature *Ae. aegypti* mosquitoes were collected during the entire sampling period. The highest numbers of immatures were found in used tyres (2124/4059; 52.33%) as compared to that of drums (1319/4059; 32.49%). Bowls showed the lowest abundance of *Aedes* immatures (616/4059; 15.18%) (Fig. 1). There was a significant difference in the abundance of immatures in the three habitat types (ANOVA, *F*_(2,__159)_ = 18.79, *P* < 0.001). Pairwise *post-hoc* Tuckeyʼs tests conducted showed that all group comparisons were statistically significant (*P* < 0.05).

### 3.3 Development time of immature stages of *Ae*. *aegypti*

The mean larval to pupal development time for immature *Ae*. *aegypti* mosquitoes was lower in tyres, 7 days as compared to bowls (9 days) and drums (12.7 days) (Kruskal-Wallis test, *H* = 7.448, *df* = 2, *P* = 0.024). A pairwise *post-hoc* Dunn test with Bonferroni adjustments showed that there was a significant difference in the mean larval to pupal development time between tyres and drums (*P* = 0.009) but no significant difference between tyres and bowls (*P* = 0.258). Furthermore, there was no significant difference in the mean larval to adult development time in males from the three habitat types (Kruskal-Wallis test, *H* = 5.728, *df* = 2, *P* = 0.06). There was a significant difference in the mean larval to adult development time of female *Ae. aegypti* mosquitoes (Kruskal-Wallis test, *H* = 6.054, *df* = 2, *P* = 0.048) (Table 2).

**Table 2:** Larval development time of immature *Aedes aegypti* mosquitoes in different habitat types.

Habitat type	Larval-pupal development time (days)	Development time of males (days)	Development time of females (days)
Tyres	7.0 ± 1.0^A^	8.0 ± 1.0^A^	8.6 ± 0.5^A^
Bowls	9.0 ± 0.0^A^	8.3 ± 0.5^A^	8.3 ± 0.5^A^
Drums	12.6 ± 1.5^B^	12.0 ± 1.0^B^	14.3 ± 1.1^B^

*Notes*: Values are means ± standard deviations. The superscript capital letters following the numerical values indicate the results of multiple comparison tests; values with the same letter were not statistically significant at *P <* 0.05 and those with different letters were statistically significant at *P* *<* 0.05.

### 3.4 Pupation rate, emergence and survivorship of immature *Ae*. *aegypti* in the different habitat types

A higher proportion of larvae pupated in bowls (0.92), compared to that of tyres (0.88) and drums (0.75) but the observed differences were not significant (Kruskal-Wallis test, *H* = 2.667, *df* = 2, *P* = 0.263). Out of the proportion of larvae that pupated, those that emerged as adults were 0.84 in tyres, 0.8 in drums and 0.77 in bowls (ANOVA, *F*_(2,__6)_ = 0.38, *P =* 0.697). The proportion of first-instar larvae that survived to adult stage was greater in tyres (0.84 ± 0.10) than in bowls (0.72 ± 0.20) and drums (0.62 ± 0.2). However, the differences in survivorship were not significant across the three habitat types (Kruskal-Wallis test, *H* = 2.822, *df* = 2, *P* = 0.238) (Table 3).

**Table 3:** Pupation rate and emergence rate of immature *Aedes aegypti* mosquitoes in different habitat types.

Habitat type	Total no. of larvae	Pupation (Mean ± SD)	Emergence (Mean ± SD)	Survivorship (Mean ± SD)
Tyres	90	0.88 ± 0.02	0.84 ± 0.10	0.84 ± 0.1^A^
Bowls	90	0.92 ± 0.17	0.77 ± 0.70	0.72 ± 0.2^A^
Drums	90	0.75 ± 0.22	0.80 ± 0.12	0.62 ± 0.2^B^

*Notes*: Values are means ± standard deviations (SD). The differences in the proportion of larvae that pupated and emerged between the three habitat types, tyres, bowls and drums were not statistically significant (*P* > 0.05). The superscript capital letters following the numerical values indicate the results of multiple comparison tests; values with the same letter were not statistically significant at *P <* 0.05 and those with different letters were statistically significant at *P* < 0.05.

**Fig. 2:**
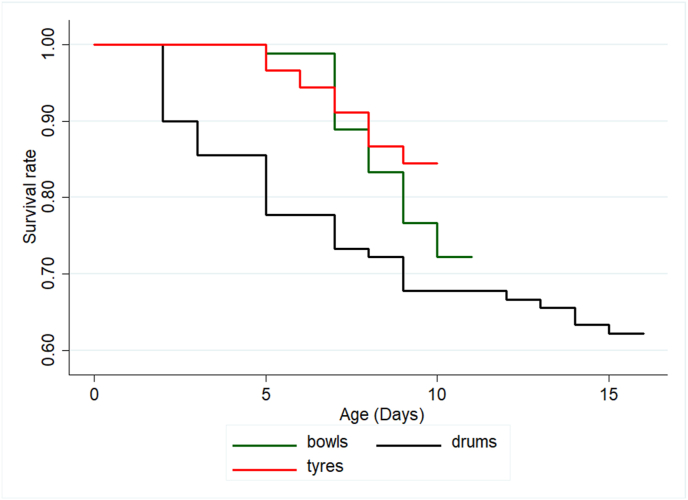
Kaplan-Meier survival curves for *Aedes aegypti* larvae in the different habitat types (tyres, bowls and drums).

Fig. 2 shows the Kaplan-Meier survivorship dynamics of *Ae*. *aegypti* in the three habitat types. The survival rate of immatures was higher in immature *Ae*. *aegypti* larvae in tyres compared to those in bowls and drums. No mortalities were observed after 9 days in car tyres, 10 days in bowls, and 15 days in drums. The log-rank test showed that all the survival curves were significantly different (*P* < 0.05 for all comparisons).

### 3.5 Sex ratio of immature *Ae*. *aegypti* mosquitoes in different habitat types

The proportion of males that emerged was higher in tyres (41.67%) and bowls (40.83%) compared to that of drums (17.50%). The proportion of emerged females was higher in drums (42.17%) compared to that of car tyres (31.33%) and bowls (26.51%) (Table 4). There was a significant difference between habitat type and the sex of emerged adults (*χ*^2^ = 15.1045, *df* = 2, *P* = 0.001). Sex ratio was male-biased in tyres (males: 65.79%; females: 34.21%) and bowls (males: 69%; females: 31%). However, in drums, more females emerged (62.5%) compared to males (37.5%).

**Table 4:** The number of males and females that emerged from the different habitat types.

Habitat type	Sample size	Males *n* (%)	Females *n* (%)	Sex ratio (Females: Males)
Tyres	76	50 (41.67)	26 (31.33)	1:1.8^A^
Bowls	71	49 (40.83)	22 (26.51)	1:1.9^A^
Drums	56	21 (17.50)	35 (42.17)	1:0.6^B^
Total	203	120 (100)	83 (100)	1:1.3

*Notes*: Frequencies are indicated by “*n*”. The superscript capital letters following the numerical values indicate the results of multiple comparison tests; values with the same letter in the sex ratio column were not statistically significant at *P <* 0.05 and those with different letters in the same column were statistically significant at *P* *<* 0.05.

### 3.6 Relationship between larval abundance, larval life-table parameters and environmental variables

Generalized Linear Mixed Model (GLMM) analysis showed that temperature and humidity had a significantly negative relationship with larval abundance in the oviposition experiments (beta-values: temperature = −56.30, humidity = −15.96, *P* < 0.05). In tyres and drums, temperature had a significantly negative influence on larval abundance (*P* < 0.05) (Supplementary Table S1). There was no significant relationship between temperature, humidity and replication on larval life-table parameters of *Ae*. *aegypti* mosquitoes (*P* > 0.05) (Supplementary Table S2).

## 4 Discussion

Understanding the ecology and biology of *Aedes* spp. mosquitoes is crucial for the control of *Aedes*-borne diseases. Development times and survivorship of various stages of mosquitoes under different environments are of particular importance, as they affect the vectorial capacity, which is tightly linked to mosquito-borne disease transmission (David et al., 2018). This study provides evidence of the oviposition preferences and larval life history traits of *Ae*. *aegypti* in different habitat types in Accra, Ghana. The findings from our study showed high larval abundance in tyres compared to that of bowls and drums. *Aedes aegypti* larvae from tyres showed a significantly shorter development time and a high survivorship compared to the other habitat types.

The immature abundance of *Ae. aegypti* was used as a proxy for oviposition preference in the three different habitat types studied. The highest larval abundance was observed in tyres, suggesting that Ghanaian *Ae. aegypti* mosquitoes prefer to breed in tyres compared to other habitat types. This finding is in line with that of Owusu-Asenso et al. (2022) where the highest densities of *Ae. aegypti* larvae were registered in car tyres (Owusu-Asenso et al., 2022). Car tyres are among the most productive aquatic habitats across West Africa (Ouattara et al., 2019; Tedjou et al., 2020; Egid et al., 2022). This may be because discarded car tyres are less prone to disturbance as compared to other habitat types such as containers, tin cans or coconut shells. Also, the internal conditions in car tyres such as reduced light and low humidity attract gravid female *Aedes* spp. mosquitoes (Dom et al., 2013). Car tyres have a narrow opening thus providing some level of shade to the immature larvae and reducing the amount of light entering the habitat (Egid et al., 2022). High mosquito densities have been associated with habitats that have some level of shading (Yee et al., 2012).

Thus, car tyres may be targeted for larval source management in the control of *Aedes* spp. Although car tyres are the most productive habitat type, discarded containers are also a common source of aquatic habitats. Drums were the second most abundant habitat in our study. A risk factor for the presence of *Aedes* spp. is the storage of water in drums for drinking or domestic use. A study from Cape Coast, Ghana found more storage containers to be infested with *Ae. aegypti* larvae in areas with water storage compared to areas with adequate access to piped water (Kudom, 2020).

This study observed that the mean development time was significantly longer in the larvae of *Ae*. *aegypti* from drums compared to that from tyres. The mean development time was similar between tyres and bowls and shorter as compared to that of drums. The short mean development time of *Aedes* spp. mosquito larvae has important epidemiological implications. Rapid larval development favours higher vector densities, which may increase disease transmission. This is because the longer development time will expose the larvae to predation and loss of habitat through desiccation (Egid et al., 2022). This negatively impacts the vectorial capacity of the vectors (Rivero et al., 2010).

There were no significant differences in the emergence of *Ae. aegypti* mosquitoes across the three habitat types. However, the proportion of emerging adults was higher in car tyres and drums compared to bowls. Larval survivorship was higher in car tyres coupled with high pupation and emergence rates. These findings suggest that car tyres may be responsible for the high densities of adult *Ae. aegypti* mosquitoes, which may facilitate arboviral transmission in Accra, Ghana. Car tyres are known to contain soluble chemicals and metals such as barium, cadmium and zinc, that gradually leach into the larval habitat water and have negative effects on larval survival of *Aedes* spp. (Wik and Dave, 2009; Villena et al., 2017). However, high densities of *Aedes* spp. larvae were associated with car tyres in our study and other studies (Ouattara et al., 2019; Tedjou et al., 2020; Owusu-Asenso et al., 2022). This suggests that local *Ae*. *aegypti* populations may have developed tolerance mechanisms to the compounds that leach from the tyres and hence further studies are needed.

Significant male bias was observed in the emerged adults from car tyres and bowls. Sex-specific responses such as responses to temperature and humidity have been associated with sex ratio imbalances (Lounibos and Escher, 2008). Male bias in *Aedes* spp. mosquitoes was found to be likely associated with year-to-year variations in temperatures (Lounibos and Escher, 2008). Another factor that has been associated with sex ratio differences is larval diet. Larvae of *Ae*. *aegypti* used for our larval life-table experiments were not provided any diet. Some studies have reported that *Ae. aegypti* larvae reared with less food produce more males whereas the sex ratio was female-skewed when the larvae were fed with more food (Puggioli et al., 2017; Gunathilaka et al., 2018). This may explain the high male bias observed in the tyres and bowls. However, in drums, more females emerged as adults than males, which may suggest that other factors may also be involved in the sex ratio differences observed.

Environmental conditions such as temperature and relative humidity affect the larval ecology of mosquitoes. The environmental temperature alters mosquito population dynamics by affecting the development of immature stages (Couret and Benedict, 2014). GLMM results from this study showed that temperature and relative humidity had a negative association with larval abundance. This suggests that higher environmental temperatures (above 32 °C) may decrease the survival and development of mosquitoes. Similar findings have been observed in other studies (Christiansen-Jucht et al., 2014; Yang et al., 2020). *Aedes aegypti* can develop at temperatures as low as 16 °C, with an upper limit of 34 °C. However, development time has been found to be shorter at higher temperatures (30 °C *vs* 21 °C) (Couret and Benedict, 2014).

Our study had several limitations. The experiments were conducted in a controlled environment thus this study did not take into account potential biological factors such as predation and competition, which may affect survivorship. The larvae were not provided any diet hence its effect on larval survival and productivity cannot be ascertained. Previous studies reported that the size of *Ae*. *aegypti* is dependent on variations in food and population density of the immature stages (Jirakanjanakit et al., 2007). Larvae of *Ae. aegypti* are recognized for their high plasticity, demonstrating the ability to undergo development under different microorganism-based diets (Souza et al., 2019). Therefore, bacteria that are found in the rainwater used may have provided a source of feed to the larvae. Lastly, the number of replicates used was small, hence the results may not be generalized. Therefore, further studies can be conducted with a higher number of replicates to provide stronger conclusions on *Ae. aegypti* larval ecology.

## 5 Conclusions

This study showed that car tyres are the preferred choice for oviposition for gravid female *Ae. aegypti* mosquitoes. Larvae of *Ae. aegypti* from car tyres showed a shorter larval development time and higher survivorship compared to other habitat types. This suggests that car tyres may be playing a significant role in the ecology of *Ae. aegypti* mosquitoes, thus facilitating arboviral transmission. This study provides baseline information that is essential for wider studies towards a better understanding of the ecology of *Ae. aegypti* to develop appropriate strategies for their control in Ghana.

## Funding

This study was supported by grants from the National Institute of Health (D43 TW 011513).

## Ethical approval

Not applicable.

## CRediT authorship contribution statement

**Anisa Abdulai:** Conceptualization, Methodology, Investigation, Data curation, Formal analysis, Writing – original draft, Writing – review & editing. **Christopher Mfum Owusu-Asenso:** Methodology, Investigation, Formal analysis, Writing – original draft, Writing – review & editing. **Christodea Haizel:** Investigation, Writing – review & editing. **Sebastian Kow Egyin Mensah:** Investigation, Writing – review & editing. **Isaac Kwame Sraku:** Investigation, Writing – review & editing. **Daniel Halou:** Investigation, Writing – review & editing. **Richard Tettey Doe:** Investigation, Writing – review & editing. **Abdul Rahim Mohammed:** Investigation, Writing – review & editing. **Yaw Akuamoah-Boateng:** Investigation, Writing – review & editing. **Akua Obeng Forson:** Conceptualization, Data curation, Supervision, Writing – review & editing. **Yaw Asare Afrane:** Conceptualization, Data curation, Methodology, Funding acquisition, Supervision, Project administration, Resources, Writing – review & editing. All authors read and approved the final manuscript.

## Declaration of competing interests

The authors declare that they have no known competing financial interests or personal relationships that could have appeared to influence the work reported in this paper.

## Data Availability

The data supporting the conclusions of this article are included within the article and its supplementary files. All datasets generated and/or analysed during this study are available on request.
