# Supplementary material for: The role of car tyres in the ecology of Aedes aegypti mosquitoes in Ghana
Source: Curr Res Parasitol Vector Borne Dis. 2024 Apr 30;5:100176. doi: 10.1016/j.crpvbd.2024.100176 (PMC11091510; doi:10.1016/j.crpvbd.2024.100176)
Supplement: Multimedia component 1 [file mmc1.pdf]

Supplementary Table S1. Generalized linear models of the influence of temperature, humidity and replication on larval abundance of *Aedes aegypti* in the oviposition experiments.

Larval abundance	Effect	Estimate ± SE	Z-value	Pr > *z*
**All habitat types**	Intercept	3029.99 ± 705.02	4.29	0.001
	Temperature	-56.30 ± 17.83	-3.16	0.002*
	Relative humidity	-15.96 ± 3.33	-4.80	0.001*
	Replicates	-8.40 ± 11.80	-0.71	0.477
**Tyres**	Intercept	5255.33 ± 1997.48	2.64	0.009
	Temperature	-214.80 ± 96.55	-2.22	0.026*
	Relative humidity	-13.40 ± 11.84	1.13	0.258
	Replicates	-46.250 ± 27.174	-1.7	0.089
**Bowls**	Intercept	1896.89 ± 963.97	1.97	0.049
	Temperature	-63.238 ± 46.59	-1.36	0.175
	Relative humidity	-1.22 ± 5.71	-0.21	0.831
	Replicates	17.50 ± 13.11	1.33	0.182
**Drums**	Intercept	3592.89 ± 1052.91	3.41	0.001
	Temperature	-121.700 ± 50.89	-2.39	0.017*
	Relative humidity	-0.929 ± 6.24	-0.15	0.880
	Replicates	7.37 ± 14.32	0.51	0.610

*Significant at $P < 0.05$

Supplementary Table S2. Generalized linear models of the influence of temperature, humidity and replication on larval life-table parameters of *Aedes aegypti*.

Larval life-table parameters	Effect	Estimate ± SE	Z-value	Pr > *z*
**Development time**	Intercept	17.29 ± 31.15	0.55	0.579
	Temperature	0.046 ± 0.60	0.07	0.939
	Relative humidity	-0.12 ± 0.38	-0.31	0.755
	Replicates	0.23 ± 1.00	0.23	0.817
**Pupation rate**	Intercept	4.88 ± 2.85	1.71	0.087
	Temperature	-0.09 ± 0.05	-1.81	0.070
	Relative humidity	-0.02 ± 0.04	-0.59	0.553
	Replicates	-0.05 ± 0.08	0.63	0.531
**Survival rate**	Intercept	127.85 ± 321.81	0.39	0.691
	Temperature	-8.30 ± 6.29	-1.32	0.187
	Relative humidity	1.71 ± 3.94	0.43	0.664
	Replicates	3.31 ± 10.51	0.31	0.753
**Emergence rate**	Intercept	-0.39 ± 1.44	-0.27	0.785
	Temperature	0.005 ± 0.03	0.16	0.871
	Relative humidity	0.015 ± 0.02	0.85	0.397
	Replicates	-0.08 ± 0.05	-1.37	0.169
